# Biocatalysis: An Eco-Friendly Scenario for the Manufacturing of APIs

**DOI:** 10.3390/ijms24054474

**Published:** 2023-02-24

**Authors:** Jesús Fernández-Lucas

**Affiliations:** 1 Applied Biotechnology Group, Biomedical Science School, Universidad Europea de Madrid, Urbanización El Bosque, Calle Tajo, s/n, 28670 Villaviciosa de Odón, Spain; jesus.fernandez2@universidadeuropea.es; 2 Grupo de Investigación en Ciencias Naturales y Exactas, GICNEX, Universidad de la Costa, CUC, Calle 58 # 55–66, Barranquilla 080002, Colombia

Nowadays, the worldwide demand for Active Pharmaceutical Ingredients (APIs) requires novel, cost-effective, safe, and environmentally friendly synthetic processes. Given this scenario, biocatalysis has started to take an ever-growing impact on industrial chemical processes [1,2]. The application of enzymes to catalyze industrial reactions has the potential to make chemical processes less contaminant and more sustainable, in accordance with the concept and principles of Green Chemistry [3]. Moreover, biocatalysis offers additional advantages, such as the possibility to perform one-pot reactions, a higher region- and stereo-selectivity, or the absence of intermediates that require tedious purification steps, which make enzyme-mediated processes a powerful ally for a successful industrial implementation of a process. 

Gone are the days when the harsh operational conditions, the narrow specificity and low promiscuity of enzymes, or the high cost of enzyme production usually hampered the industrial applications of enzymes for APIs manufacturing. In contrast, recent advances in biocatalysis lead to a breeding ground to satisfy industry requirements [4]. 

This Special Issue will cover some recent and relevant findings concerning the enzyme-mediated manufacturing of APIs. To this end, a total of seven articles, including five experimental articles and two revisions, were compiled to show the state of the art.

Due to the harsh conditions commonly used in industry, biocatalyst stabilization is an essential attribute for industrial implementation. In this respect, different experimental articles address this topic. In the first article, Mao and coworkers [5] evaluate the characteristics of different polymers as crosslinking agents to improve the stability and catalytic properties of *Thermomyces lanuginosus* lipase adsorbed onto hydrophobic supports. In a similar vein, Di Fabio et al. [6] display a novel biocatalyst for the oxidative deamination of a wide range of primary amines into the corresponding aldehydes based on the covalent immobilization of amino oxidase from *Lathyrus cicero* onto magnetic microparticles. 

The transglycosylation reaction catalyzed by nucleoside phosphorylases (NPs) [7] or 2’-deoxyribosyltransferases (NDTs) [2] has proven to be a versatile tool for the synthesis of a wide spectrum of nucleoside analogs. To this end, an engineered NDT from *Lactobacillus delbrueckii* has been designed by Cruz et al. [8] by prediction of disulfide bond engineering sites. Additionally, this new thermostable variant was successfully employed as a catalyst for the enzyme-mediated production of nelarabine. Similarly, Drenichev et al. [9] performed a comparative analysis of the transglycosylation conditions catalyzed by different *E. coli* NPs. As a consequence, different ribonucleoside analogs were synthesized at different experimental conditions through a two-step reaction catalyzed by different purine and pyrimidine NPs. In another contribution, Cruz et al. [10] explored the viability of a magnetic multi-enzymatic system for cladribine production, based on the sequential action of *Leismanhia mexicana* type I NDT (*Lm*PDT) and hypoxanthine phosphoribosyltransferase from *E. coli* (*Ec*HGPRT) immobilized onto magnetic microspheres.

Following this tendency, Siedentop and Rosenthal [11] present an exhaustive revision of the industrial implementation of enzymatic cascades for API manufacturing. Moreover, the authors pay special attention to multi-enzymatic reactions for API synthesis that are close to an industrial application, including economic and ecological to assess their environmental friendliness and applicability. 

Last but not least, Godoy et al. highlight the importance of biocatalyst design and process engineering to assess the potential of microbial lipases as biocatalysts for the production of pharmaceutical building blocks [12]. The authors focused their work on the comprehension of the structure–function relationship of microbial lipases but also included detailed comments about recent trends in the immobilization of lipases. Finally, a comprehensive description of microbial lipase application development toward a greener industry was presented.

We hope that the advances presented in this Special Issue will be of interest to the readers. We thank all the authors, reviewers, and Editorial Board members of *International Journal of Molecular Sciences*, whose contributions and support were vital to the publication of this Special Issue.
